# Distribution of ocular biometric parameters and optimal model of anterior chamber depth regression in 28,709 adult cataract patients in China using swept‐source optical biometry

**DOI:** 10.1186/s12886-021-01932-4

**Published:** 2021-04-13

**Authors:** Qiong Lei, Haixia Tu, Xi Feng, Julio Ortega-Usobiaga, Danmin Cao, Yong Wang

**Affiliations:** 1 Aier Eye Hospital of Wuhan University, Wuhan, China; 2 Aier School of Ophthalmology, Central South University, Changsha, China; 3 Department of Cataract and Refractive Surgery, Cl í nica Baviera-AIER Eye Hospital Group, Bilbao, Spain

**Keywords:** Axial length, Anterior chamber depth, Lens thickness, Cataract, IOLMaster 700

## Abstract

**Background:**

The purpose of this study was to evaluate the ocular biometric parameters in adult cataract patients from China and create an anterior chamber depth (ACD) regression model.

**Methods:**

The ocular biometric records of 28,709 right eyes of cataract surgery candidates who were treated at Aier Eye Hospitals in nine cities from 2018 to 2019 were retrospectively analyzed. All measurements were taken with IOLMaster 700. We included patients who were at least 40 years old and were diagnosed with cataract.

**Results:**

The mean age of the patients was 68.6 ± 11.0 years. The mean values recorded were as follows: axial length (AL), 24.17 ± 2.47 mm; mean keratometry (Km) value, 44.26 ± 1.70 D; corneal astigmatism (CA), 1.06 ± 0.96 D; ACD, 3.02 ± 0.45 mm; lens thickness (LT), 4.52 ± 0.45 mm; central corneal thickness (CCT), 0.534 ± 0.04 mm; and white to white (WTW) corneal diameter, 11.64 ± 0.46 mm. ACD correlated positively with AL (Spearman coefficient, 0.544) and WTW (0.300), but negatively with LT (-0.660) and age (-0.285) (all *P* < 0.01). In the multivariate regression analysis of ACD, which included LT, AL, WTW, sex, Km, CCT, and age, there was a reasonable prediction with adjusted *R*^2^ = 0.641.

**Conclusions:**

Cataract patients with longer AL and wider WTW have deeper ACD. With increasing age and lens thickening ACD becomes shallower. Based on the standardized coefficients of ACD multivariate regression analysis from the study, LT is the main factor that affects ACD, and is followed by AL.

## Background

Modern cataract surgery has transformed from a rehabilitative procedure to a refractive operation. This can be attributed to the development of measuring devices, lens calculation formulas, surgical technology, and intraocular lenses. Accurate prediction of refractive outcomes after cataract surgery is crucial. When biometry is based on optical coherence interferometry (Zeiss IOLMaster), the error from anterior chamber depth (ACD) prediction amounts to approximately 42 % of the total refractive prediction error [1]. Olsen [2] found that postoperative ACD is significantly predictable by a five-variable regression method that incorporates preoperative axial length (AL), ACD, keratometry (K), lens thickness (LT), and refraction. Therefore, it is essential to acquire the biometric characteristics of cataractous eyes to enable the utilization of precise ocular biometric parameters for accurate intraocular lens calculation.

Previous research has confirmed that female sex, older age, shorter AL, shallow ACD, and smaller LT were risk factors for angle closure [3]. These risk factors are also closely associated with elderly cataract patients.

To the best of our knowledge, there is no study of a large series of biometry measurements acquired with the IOLMaster 700 device in the existing literature. The purpose of this study was to review and evaluate their biometric parameters and create an ACD regression model for cataract surgery candidates aged 40 years or older. The results of this study may provide a new reference for cataract patients based on a large group of patients.

## Methods

### Study population

The medical records of 28,709 cataract surgery candidates over a year period (October 2018 to October 2019) (11,753 men [40.9 %] and 16,956 women [59.1 %]) from the Aier Eye Hospitals in nine provincial capitals in China were reviewed retrospectively. Due to the retrospective nature of the study, the need for informed consent was waived. The retrospective study was approved by the Ethics Committee of Aier Eye Hospital and was performed in accordance with the Declaration of Helsinki.

The ocular parameters were measured with a non-contact swept-source optical biometer (IOLMaster 700, Carl Zeiss Meditec AG, Jena, Germany). The inclusion criteria were as follows: (1) patients diagnosed with cataracts; (2) patients who were 40 years or older; and (3) patients with good quality measurements with the IOLMaster 700. The exclusion criteria were as follows: (1) patients who had complications with other eye diseases including corneal diseases, retinal diseases, glaucoma or inflammatory eye diseases; (2) patients who had a history of trauma or ocular surgery; and (3) patients who had used drugs that affected anterior chamber depth and pupil diameter, such as atropine or pilocarpidine eye drops.

### Device and measurement

**Fig. 1:**
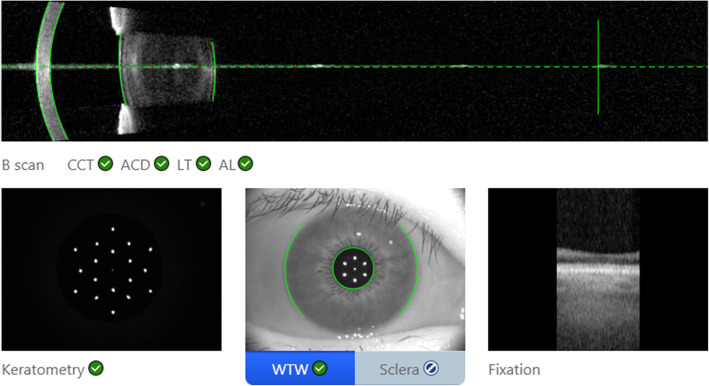
IOL Master 700 can obtain OCT images of the macular and visualizes the measurement of the AL

The IOLMaster 700 has a swept source optical coherence tomography (SS-OCT) scanning capacity with 1,050 nm laser infrared light and measures AL, ACD, LT, and central corneal thickness (CCT). The device can obtain OCT images of the macula and visualizes the measurement of the AL of the eye (Fig. 1). The IOL Master 700 uses telecentric keratometry for K measurements. Corneal power is measured in two meridians: the greatest and least radii of curvature (K_1_, K_2_). Corneal astigmatism (CA) is calculated as the absolute difference between K_1_ and K_2_ values. Mean keratometry (Km) is the average of K_1_ and K_2_. It provides keratometry measurements in the central 2.5 mm zone and uses a refractive index of 1.3375 for the biometry parameters. ACD is measured from the corneal epithelium to the anterior lens surface [4]. A single capturing process can provide multiple measurements for each parameter. We controlled the quality of the measurements according to the manufacturer instructions. All patients were tested by experienced examiners. The compound values of AL, ACD and LT are derived from the average of the six measurements. The compound values of K, CCT and WTW are derived from the average of the three measurements. We selected measurements with a standard deviation of less than 20 microns.

### Statistical analysis

To avoid correlation between the right and left eyes, only the right eye of each subject was used for statistical analyses. Data were processed with the SPSS software version 19.0 (IBM, USA). We performed a four-step analysis. First, we stratified the parameters according to sex and age groups. The study participants were classified into 5 groups based on age (40–49 years, 50–59 years, 60–69 years, 70–79 years, over 80 years). All eyes were divided into five groups based on AL; they were also categorized into five groups based on ACD. Second, the Mann-Whitney U Test was used to compare the ocular parameters according to sex and AL group. The chi-square test was used to compare the frequency distribution. Third, we evaluated the association between age and other ocular parameters using Spearman’s correlation. Finally, we performed a multivariate analysis with ACD as the dependent parameter and all other parameters that were significantly associated with ACD as independent variables.

Variation inflation factors were calculated to investigate multicollinearity. All *P*-values were two-sided and considered statistically significant when the values were less than 0.05.

## Results

**Fig. 2:**
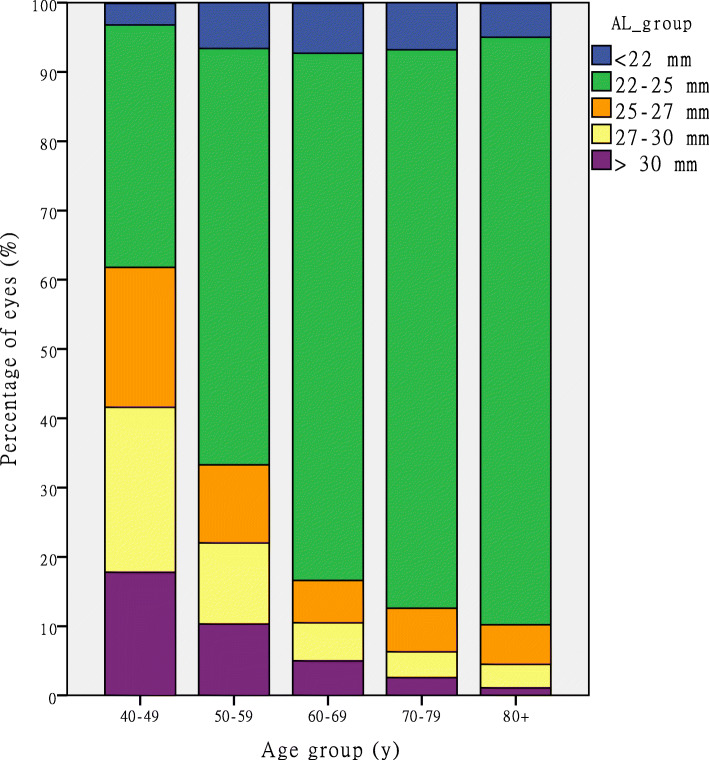
Stacked histogram comparing the percentage of eyes in different AL groups by age groups. AL = axial length

A total of 28,709 patients, including 11,753 men (40.9 %) and 16,956 women (59.1 %) diagnosed of cataract, were selected according to our inclusion and exclusion criteria. The mean age of the patients was 68.6 ± 11.0 years (range, 40–101 years). The mean values measured were as follows: AL, 24.17 ± 2.47 mm; mean keratometry (Km) value, 44.26 ± 1.70 D; corneal astigmatism (CA), 1.06 ± 0.96 D; ACD, 3.02 ± 0.45 mm; lens thickness (LT), 4.52 ± 0.45 mm; central corneal thickness (CCT), 0.534 ± 0.04 mm; and white to white (WTW) corneal diameter, 11.64 ± 0.46 mm. The details of the ocular characteristics of the right eyes stratified according to age groups are shown in Table 1. The subgroups and the distribution of AL and ACD are shown in Table 2. Figure 2 illustrates the percentage of patients in the AL groups stratified according to age. The proportion of patients with long AL (AL > 25 mm) decreased with age.

**Table 1:** Distribution of the ocular biometric parameters by age group

Parameter	Total	Age group (years)				
		**40–49**	**50–59**	**60–69**	**70–79**	**80+**
n (%)	28,709	1932 (6.7 %)	3658 (12.7 %)	8720 (30.4 %)	9888 (34.4 %)	4511 (15.7 %)
AL (mm)	24.17 ± 2.47	26.63 ± 3.40	24.98 ± 3.20	24.03 ± 2.39	23.75 ± 1.93	23.66 ± 1.58
Km (D)	44.26 ± 1.70	43.72 ± 2.17	44.08 ± 1.81	44.30 ± 1.61	44.38 ± 1.63	44.32 ± 1.59
CA (D)	1.06 ± 0.96	1.14 ± 0.93	1.01 ± 0.94	0.94 ± 0.88	1.08 ± 0.99	1.26 ± 1.04
ACD (mm)	3.02 ± 0.45	3.38 ± 0.41	3.21 ± 0.43	3.03 ± 0.44	2.95 ± 0.44	2.88 ± 0.41
LT (mm)	4.52 ± 0.45	4.17 ± 0.38	4.32 ± 0.43	4.50 ± 0.44	4.60 ± 0.43	4.70 ± 0.43
CCT (mm)	0.534 ± 0.04	0.533 ± 0.04	0.537 ± 0.04	0.534 ± 0.03	0.533 ± 0.04	0.533 ± 0.03
WTW (mm)	11.64 ± 0.46	11.83 ± 0.49	11.74 ± 0.46	11.63 ± 0.45	11.58 ± 0.45	11.60 ± 0.44

*AL *axial length; *Km *mean keratometry; *CA *corneal astigmatism; *ACD *anterior chamber depth; *LT *lens thickness; *CCT *central corneal thickness; *WTW *white to white

**Table 2:** Distribution of ocular biometry parameters

Parameter	Number (%)
**AL group**	
< 22 mm	1840 (6.4%)
22–25 mm	21309 (74.2%)
25–27 mm	2221 (7.7%)
27–30 mm	1879 (6.5%)
≥ 30 mm	1460 (5.1%)
**ACD group**	
< 2.0 mm	413(1.4%)
2.0–2.5 mm	3000 (10.4%)
2.5–3.0 mm	9752 (34%)
3.0–3.5 mm	11223 (39.1%)
> 3.5 mm	4321 (15.1%)

*AL* axial length; *ACD* anterior chamber depth

In this study population, the AL was statistically significantly longer in male subjects than in female subjects (24.39 mm versus 24.02 mm, *P* < 0.001). The ACD was significantly deeper in men than in women (3.12 mm versus 2.96 mm, *P* < 0.001). Men had a thicker LT than women (4.53 mm versus 4.52 mm, *P* < 0.05). The Km was flatter in men than in women (43.75 D versus 44.62 D, *P* < 0.001). The WTW was significantly wider in men than in women (11.78 mm versus 11.54 mm, *P* < 0.001). The above results were shown in Table 3.

**Table 3:** Matrix of Spearman correlation of biometric parameters

Parameter	Age	AL	ACD	LT	Km	WTW
Age	-	-0.180**	-0.285**	0.312**	0.059**	-0.133**
AL	-	-	0.544**	-0.212**	-0.423**	0.325**
ACD	-	-	-	-0.660**	-0.077**	0.300**
LT	-	-	-	-	0.014*	-0.047**
Km	-	-	-	-		-0.441**

*AL *axial length; *ACD* anterior chamber depth; *LT  *lens thickness; *Km* mean keratometry; *WTW *white to white

* *P* < 0.05

**P < 0.01

**Fig. 3:**
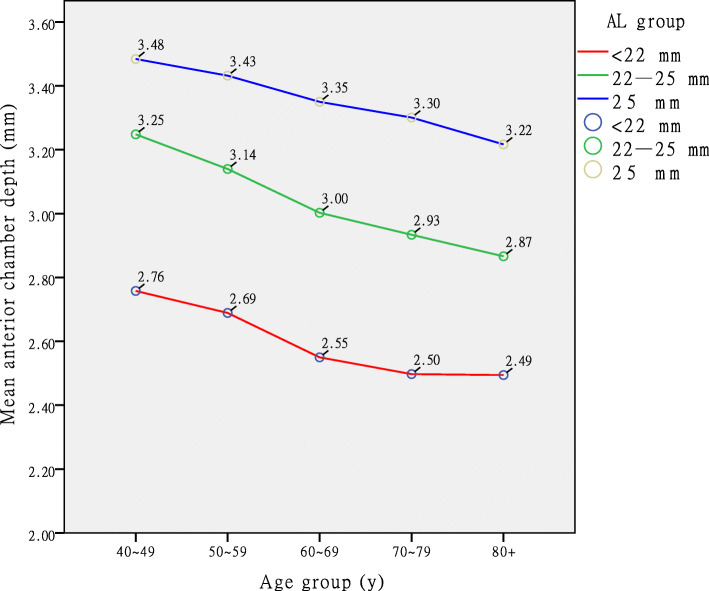
Distribution of anterior chamber depth (ACD) across age groups with data markers stratified according to three axial length (AL) groups. The ACD tended to be shallower in older patients and shorter eyes

We also compared the ACD of the three AL groups (< 22 mm, 22–25 mm and ≥ 25 mm) and found that the differences between them were statistically significant (*P* < 0.001). The ACD of the patients in the group with AL < 22 mm was the shallowest (Fig. 3).

Spearman correlation analysis revealed that age, AL, ACD, LT, Km, and WTW were all significantly correlated with each other (*P* < 0.05). ACD correlated positively with AL (*r* = 0.544, *P* < 0.01) and WTW (*r* = 0.300, *P* < 0.01), but negatively with LT (*r* = -0.660, *P* < 0.01) and age (*r* = -0.285, *P* < 0.01). ACD was highly correlated with LT and AL. The complete matrix of the correlation is presented in Table 3.

Variation inflation factors for variables were below 2, indicating that there was no clear evidence of multicollinearity. In the multivariate regression analysis of the final model, which included LT, AL, WTW, Km, sex, CCT, and age, there was a reasonable prediction with adjusted *R*^2^ = 0.641 (Table 4).

**Table 4:** Optimal model coefficients of anterior chamber depth regression

Model	Unstandardized Coefficients		Standardized B	t	Sig.
	B	Std. Error			
(Constant)	-0.522	0.091		-5.746	< 0.001
LT	-0.623	0.004	-0.621	-166.028	< 0.001
AL	0.064	0.001	0.348	91.727	< 0.001
WTW	0.249	0.004	0.251	62.288	< 0.001
Sex	-0.121	0.003	-0.131	-35.275	< 0.001
Km	0.040	0.001	0.150	37.063	< 0.001
CCT	0.471	0.046	0.037	10.180	< 0.001
Age	0.001	0.000	0.030	7.825	< 0.001

*ACD* anterior chamber depth; *LT *lens thickness; *AL* axial length; *WTW* white to white; *Km* mean keratometry; *CCT* central corneal thickness; *Std.* standard

## Discussion

We reviewed and evaluated the biometry parameters of cataract surgery candidates and created an ACD regression model for predicting the refractive outcomes of cataract surgery. Optical biometry has been well accepted as the gold standard since the introduction of the IOLMaster optical biometer in 1999 [5]. As a newly available swept-source OCT-based optical biometry device, the IOLMaster 700 provides OCT imaging of the macula and visualizes the measurement of the AL of the eye. The repeatability and reproducibility of the swept-source optical biometer is excellent and its agreement with a standard biometer is very high; better lens penetration ability and more accurate AL measurements are obtained with the swept-source biometer than with the standard biometer [6]. The SS-OCT biometer showed better penetration in dense posterior subcapsular cataracts, measuring AL successfully in 96 % of cases which possibly was a result of the reduced scattering and attenuation from ocular opacities by the 1055 nm light source used [7]. In the present study, ocular biometry was performed with the IOLMaster 700, which can capture all parameters in a single process. It is convenient for evaluating patients, especially elderly patients.

A gradual descending trend in average age was reported in five studies of patients who underwent cataract surgery (Table 5) [8–12]. To minimize selection bias, we compared the proportion of patients under 70 years of age in our study and with those of Cui’s [10] studies, which represented the cataract surgery scenario in China 10 years ago (July 2007 and June 2011). Both studies included cataract candidates scheduled for phacoemulsification, most of whom came from the urban area. The result, which indicated that there was a higher ratio of cataract patients under 70 years of age currently than there was 10 years ago, was statistically significant (χ^2^ = 199.008, *P* < 0.001). Over the last 10 years, the development of phacoemulsification and femtosecond laser-assisted cataract surgery [13, 14] and the popularity of functional intraocular lenses [15, 16] have advanced cataract surgery in China, leading to better visual outcomes. Urban Chinese cataract patients in the present study tended to choose surgical treatment earlier than cataract patients did 10 years ago, a decision which could improve their quality of life.

**Table 5:** Mean value of biometric parameters published in previous studies

Study* (Date)	Country	Method	Eyes (n)	Age (y)	AL (mm)	ACD (mm)	LT (mm)
Our study	China	IOLMaster 700	28,709	68.6 ± 11.0	24.17 ± 2.47	3.02 ± 0.45	4.52 ± 0.45
Ferreira [5] (2017)	Portugal	Lenstar	13,012	69 ± 10	23.87 ± 1.55	3.25 ± 0.44	4.32 ± 0.49
Cui [6] (2014)	China	IOLMaster	6750	70.4 ± 10.5	24.07 ± 2.14	3.01 ± 0.57	-
Jivrajka [7] (2008)	USA	US immersion	795	74.37 ± 8.93	23.46 ± 1.03	2.96 ± 0.45	4.93 ± 0.56
Hoffer [8] (1993)	USA	US contact	600	69 ± 12	23.65 ± 1.28	-	4.63 ± 0.68
Hoffer [9] (1980)	USA	US contact	7500	72 ± 10	23.65 ± 1.35	3.24 ± 0.44	-

* First Author

*US* A-scan ultrasound biometry; *AL* axial length; *ACD* anterior chamber depth; *LT* lens thickness

In present study, more female patients scheduled cataract surgery than male patients (59.1 % versus 40.9 %). This finding is almost identical to those of previous published studies [9, 17] (60 % versus 40 %). Furthermore, female patients presented for surgery at a slightly older age than male patients (mean: 68.8 years versus 68.4 years, *P* = 0.005). This is similar to the results of Jivrajka’s report (mean: 75 years versus 73 years), which considered that women had a longer life expectancy than men [9]. Moreover, some studies showed that women exhibit a higher prevalence of some types of cataract than men [18, 19]. Freeman [20] suggested that a potentially modifiable factor in cataractogenesis may be a woman’s exposure to postmenopausal estrogen. This partially explains the sex-related differences in the rates of cataract surgery. Moreover, we believe that elderly women are more concerned about their eye health and are more likely to undergo surgery than elderly men. This needs to be confirmed by further research.

In the present study, female patients presented for surgery with a shorter average AL (mean 24.02 mm versus 24.39 mm, *P* < 0.001) and shallower ACD (mean 2.96 mm versus 3.12 mm, *P* < 0.001) than male patients. This echoes the findings of other studies of cataract patients in different countries [21–23]. Some studies showed that body height is positively correlated with AL and ACD; taller persons tended to have longer AL and deeper ACD than shorter people, and men are generally taller than women [9, 24, 25]. The sex-related differences in the AL and ACD measured in the present study may be attributed to the association between ocular dimensions and stature. Further, this is consistent with the higher incidence of primary angle closure (PAC) in women than in men [3, 26]. Research confirms that the female sex, older age, shorter AL, shallow ACD, and larger LT are risk factors for angle closure [3, 26]. These risk factors are closely associated with elderly cataract patients.

In the present study, the mean AL was 24.17 ± 2.47 mm; this result is similar to those of previous reports by Yu [27] (24.38 ± 2.47 mm) and Huang [28] (24.32 ± 2.42 mm). We found that AL was negatively correlated with age (*r* = -0.180 *P* < 0.001) (Table 4). The longest mean AL was in the 40–49 years age group (26.63 ± 3.40 mm); this mean AL is longer than that of Cui’s report (25.97 ± 3.53 mm) [10]. This may be because our study population had a higher proportion of eyes with a longer AL (AL > 26 mm) than the population in Cui’s study [10] (14.7 % versus 11.9 %). This is especially apparent in the 40–49 years age group in our study, in which the proportion was as high as 52.1 %. The proportion of eyes with AL > 25 mm decreased with age (Fig. 2). This corroborates Jivrajka’s supposition that high myopia with increased AL predisposes to the development of cataract at a younger age [9]. On the contrary, newer lens formulas and adjustments/transformations are now available to reduce postoperative predictive error in high myopes [29, 30]. Furthermore, the levels of satisfaction and spectacle independence experienced by high myopes were reasonably high after implantation of multifocal or trifocal intraocular lens [15, 31]. Since these outcomes enhance the confidence of high myopes, they are more inclined to schedule cataract surgery at a younger age.

In the present study, the proportion of eyes with short AL (AL < 22 mm) was 6.4 %, which is similar to that of other studies [10, 28]; the proportion of eyes with shallow ACD (ACD < 2.5 mm) was 11.8 %. The refractive status of a patient with a shallow ACD and a short AL after cataract surgery would tend toward a myopic shift. Conversely, a patient with deep ACD and long AL would move toward a hyperopic shift, which was related to the postoperative ACD change [32]. The relative change in ACD after phacoemulsification is larger in short eyes than in normal and long eyes [33]. Melles found that the Barrett Universal Formula had the lowest mean absolute prediction error for eyes with short AL and shallow ACD [34]. This guided us toward choosing the suitable formula for these kinds of patients.

The mean ACD in the present study was 3.02 ± 0.45 mm. From the matrix of Spearman correlation of biometric parameters (Table 3) cataract patients with longer AL and wider WTW have deeper ACD. With increasing age and lens thickening ACD becomes shallower. The mean LT was 4.52 ± 0.45 mm and tended to be thicker in older patients and in shorter eyes. These findings are consistent with the findings of other studies [8, 9, 35]. These results confirm Hoffer’s [8] mechanism of emmetropization: the lens thins (or decreases in power) as the eye gets longer (myopic) and thickens (or increases in power) as the eye gets shorter (hyperopic). The Km in the present study was 44.26 ± 1.70 D and tended to be greater in shorter eyes, which is also in line with the mechanism of emmetropization.

Based on the standardized coefficients of ACD multivariate regression analysis, LT is the main factor that affects ACD, and is followed by AL (Table 4). Adjusted *R*^2^ = 0.641 means that 64.1 % of the variance could be explained by this model. A study of an elderly Chinese population showed that approximately one in five people aged 50 years and over developed some form of angle closure over a 10-year period, and reported best cut-off values of 2.60, 4.72, and 22.92 mm for ACD, LT, and AL, respectively, in predicting incident PAC [26]. In the present study, the mean ACD of patients in the 60–69 age group was 2.55 mm when the AL was < 22 mm (Fig. 3), and it tended to be shallower with age. Therefore, cataract patients with AL < 22 mm should be more concerned about their intraocular pressure when they are over 60 years old.

This study has certain limitations. First, the data from the nine hospitals do not completely represent the ocular parameters of the overall population in China. Second, we need to collect more information about anthropometric characteristics, education, occupation, income, and the type of intraocular lens, and evaluate the correlations among them. We will conduct more research to improve on the findings of the analyses.

In summary, we collated data on the ocular biometric parameters of cataract surgery candidates in China, compared them, analyzed their correlations, and created an ACD regression model. This study provided reference values for AL, Km, CA, ACD, LT, CCT and WTW by using IOLMaster 700 for adult cataract patients in China. Cataract patients with longer AL and wider WTW have deeper ACD. With increasing age and lens thickening ACD becomes shallower. Based on the standardized coefficients of ACD multivariate regression analysis from the study, LT is the main factor that affects ACD, and is followed by AL.

## Data Availability

The datasets used and/or analysed during the current study are available from the corresponding author on reasonable request.

## Abbreviations

ACD: Anterior chamber depth
AL: Axial length
K: Keratometry
LT: Lens thickness
SS-OCT: Swept source optical coherence tomography
CCT: Central corneal thickness
CA: Corneal astigmatism
Km: Mean keratometry
WTW: White to white

## References

[1] Olsen T (2007). Calculation of intraocular lens power: a review. Acta Ophthalmol Scand.

[2] Olsen T (2006). Prediction of the effective postoperative (intraocular lens) anterior chamber depth. J Cataract Refract Surg.

[3] Schuster AK, Pfeiffer N, Nickels S, Schulz A, Hohn R, Wild PS, Binder H, Munzel T, Beutel ME, Vossmerbaeumer U (2016). Distribution of Anterior Chamber Angle Width and Correlation With Age, Refraction, and Anterior Chamber Depth-The Gutenberg Health Study. Invest Ophthalmol Vis Sci.

[4] Hoffer KJ (2011). Definition of ACD. Ophthalmology.

[5] Santodomingo-Rubido J, Mallen EA, Gilmartin B, Wolffsohn JS (2002). A new non-contact optical device for ocular biometry. Br J Ophthalmol.

[6] Srivannaboon S, Chirapapaisan C, Chonpimai P, Loket S (2015). Clinical comparison of a new swept-source optical coherence tomography-based optical biometer and a time-domain optical coherence tomography-based optical biometer. J Cataract Refract Surg.

[7] Kurian M, Negalur N, Das S, Puttaiah NK, Haria D, Thakkar JTS. MM: Biometry with a new swept-source optical coherence tomography biometer: Repeatability and agreement with an optical low-coherence reflectometry device. J Cataract Refract Surg. 2016;42(4):577–81. 10.1016/j.jcrs.2016.01.038 27113881

[8] Hoffer KJ (1993). Axial dimension of the human cataractous lens. Arch Ophthalmol.

[9] Jivrajka R, Shammas MC, Boenzi T, Swearingen M, Shammas HJ (2008). Variability of axial length, anterior chamber depth, and lens thickness in the cataractous eye. J Cataract Refract Surg.

[10] Cui Y, Meng Q, Guo H, Zeng J, Zhang H, Zhang G, Huang Y, Lan J (2014). Biometry and corneal astigmatism in cataract surgery candidates from Southern China. J Cataract Refract Surg.

[11] Ferreira TB, Hoffer KJ, Ribeiro F, Ribeiro P, O’Neill JG (2017). Ocular biometric measurements in cataract surgery candidates in Portugal. PLoS One.

[12] Hoffer KJ (1980). Biometry of 7,500 cataractous eyes. Am J Ophthalmol.

[13] Chen X, Yu Y, Song X, Zhu Y, Wang W, Yao K (2017). Clinical outcomes of femtosecond laser-assisted cataract surgery versus conventional phacoemulsification surgery for hard nuclear cataracts. J Cataract Refract Surg.

[14] Zhang X, Yu Y, Zhang G, Zhou Y, Zhao G, Chen M, Wang Y, Zhu S, Zhang H, Yao K (2019). Performance of femtosecond laser-assisted cataract surgery in Chinese patients with cataract: a prospective, multicenter, registry study. BMC Ophthalmol.

[15] Wang Q, Zhao G, Wang Q, Jia W (2012). Visual quality after AcrySof IQ ReSTOR intraocular lens implantation in eyes with high myopia. Eur J Ophthalmol.

[16] Xu Z, Cao D, Chen X, Wu S, Wang X, Wu Q: Comparison of clinical performance between trifocal and bifocal intraocular lenses: A meta-analysis. *PLoS One* 2017, 12(10):e0186522. 10.1371/journal.pone.0186522 PMC5657996 29073156

[17] Shammas HJ, Shammas MC (2015). Measuring the cataractous lens. J Cataract Refract Surg.

[18] Tsai S-Y, Hsu W-M, Cheng C-Y, Liu J-H, Chou P (2003). Epidemiologic study of age-related cataracts among an elderly chinese population in Shih-Pai, Taiwan. Ophthalmology.

[19] Xu L, Cui T, Zhang S, Sun B, Zheng Y, Hu A, Li J, Ma K, Jonas JB (2006). Prevalence and risk factors of lens opacities in urban and rural Chinese in Beijing. Ophthalmology.

[20] Freeman EE, Munoz B, Schein OD, West SK (2001). Hormone replacement therapy and lens opacities: the Salisbury Eye Evaluation project. Archives of ophthalmology.

[21] Shufelt C, Fraser-Bell S, Ying-Lai M, Torres M, Varma R (2005). Los Angeles Latino Eye Study G: **Refractive error, ocular biometry, and lens opalescence in an adult population: the Los Angeles Latino Eye Study**. Invest Ophthalmol Vis Sci.

[22] PC H, WW H (2010). Analysis of biometry and prevalence data for corneal astigmatism in 23,239 eyes. J Cataract Refract Surg.

[23] JJ RF, IG WGB, TY MKR. W, P M: Distribution of axial length and ocular biometry measured using partial coherence laser interferometry (IOL Master) in an older white population. Ophthalmology. 2010;117(3):417–23. 10.1016/j.ophtha.2009.07.028 20031227

[24] Nangia V, Jonas JB, Sinha A, Matin A, Kulkarni M, Panda-Jonas S (2010). Ocular axial length and its associations in an adult population of central rural India: the Central India Eye and Medical Study. Ophthalmology.

[25] Wei S, Sun Y, Li S-M. Jian-Ping Hu, Kai Cao, Wenzai An: Effect of body stature on refraction and ocular biometry in Chinese young adults: The Anyang University Students Eye Study. Clinical Experimental Optometry. 2020;104(2):201–6. 10.1111/cxo.13137 32869355

[26] Wang L, Huang W, Huang S, Zhang J, Guo X, Friedman DS, Foster PJ, He M (2019). Ten-year incidence of primary angle closure in elderly Chinese: the Liwan Eye Study. Br J Ophthalmol.

[27] Yu JG, Zhong J, Mei ZM, Zhao F, Tao N, Xiang Y (2017). Evaluation of biometry and corneal astigmatism in cataract surgery patients from Central China. BMC Ophthalmol.

[28] Huang Q, Huang Y, Luo Q, Fan W (2018). Ocular biometric characteristics of cataract patients in western China. BMC Ophthalmol.

[29] Chong EW, Mehta JS (2016). High myopia and cataract surgery. Curr Opin Ophthalmol.

[30] Wang L, Koch DD (2018). Modified axial length adjustment formulas in long eyes. J Cataract Refract Surg.

[31] Steinwender G, Schwarz L, Bohm M, Slavik-Lencova A, Hemkeppler E, Shajari M, Kohnen T (2018). Visual results after implantation of a trifocal intraocular lens in high myopes. J Cataract Refract Surg.

[32] Ning X, Yang Y, Yan H, Zhang J (2019). Anterior chamber depth - a predictor of refractive outcomes after age-related cataract surgery. BMC Ophthalmol.

[33] Muzyka-Wozniak M, Ogar A (2016). Anterior chamber depth and iris and lens position before and after phacoemulsification in eyes with a short or long axial length. J Cataract Refract Surg.

[34] Melles RB, Holladay JT, Chang WJ (2018). Accuracy of Intraocular Lens Calculation Formulas. Ophthalmology.

[35] Hoffer KJ (1993). The Hoffer Q formula: A comparison of theoretic and regression formulas. Journal of Cataract Refractive Surgery.

